# Molecular characterization of ‘*Candidatus* Borrelia tachyglossi’ (family *Spirochaetaceae*) in echidna ticks, *Bothriocroton concolor*

**DOI:** 10.1099/ijsem.0.001929

**Published:** 2017-05-05

**Authors:** Siew-May Loh, Amber Gillett, Una Ryan, Peter Irwin, Charlotte Oskam

**Affiliations:** ^1^​ Vector and Water-Borne Pathogen Research Group, School of Veterinary and Life Sciences, Murdoch University, Perth, Western Australia, Australia; ^2^​ Australia Zoo Wildlife Hospital, Beerwah, Queensland, Australia

**Keywords:** Australian ticks, *Candidatus* Borrelia tachyglossi, Spirochaetaceae, *Bothriocroton concolor*, Echidna

## Abstract

Recently, a novel species of the genus *Borrelia*was identified in *Bothriocroton concolor* and *Ixodes holocyclus* ticks from echidnas. Analyses of 16S rRNA and *flaB* genes identified three closely related genotypes of this bacterium (*Borrelia* sp. Aus A-C) that were unique and distinct from previously described borreliae. Phylogenetic analyses of *flaB* (763 bp), *groEL* (1537 bp), *gyrB* (1702 bp) and *glpQ* (874 bp) gene sequences and concatenated sequences (3585 bp) of three gene loci (16S rRNA, *flaB* and *gyrB*) were consistent with previous findings and confirm that this novel species of the genus *Borrelia* is more closely related to, yet distinct from, the Reptile-associated (REP) and Relapsing Fever (RF) groups. At the *flaB* locus, genotypes A, B and C shared the highest percentage sequence similarities (87.9, 88 and 87.9 %, respectively) with *B.orrelia turcica* (REP), whereas at the *groEL* and *gyrB* loci, these genotypes were most similar (88.2–89.4 %) to *B.orrelia hermsii* (RF). At the *glpQ* locus, genotypes A and B were most similar (85.7 and 85.4 % respectively) to *Borrelia* sp. Tortoise14H1 (REP). The presence of the *glpQ* gene, which is absent in the Lyme Borreliosis group spirochaetes, further emphasises that the novel species of the genus *Borrelia* characterized in the present study does not belong to this group. Phylogenetic analyses at multiple loci produced consistent topographies revealing the monophyletic grouping of this bacterium, therefore providing strong support for its species status. We propose the name ‘*Candidatus* Borrelia tachyglossi’, and hypothesize that this species of the genus *Borrelia* may be endemic to Australia. The pathogenic potential of this bacterium is not yet known.

The family *Spirochaetaceae* is classified under the order *Spirochaetales*, belonging to the phylum *Spirochaetes*. This family consists of genera that are of concern to human health, such as *Borrelia* and *Treponema* [1], with common pathogenic species including ‘*Treponema pallidum* subsp. *pallidum*', the causative agent of syphilis worldwide [2], and ‘*Treponema pallidum* subsp. *pertenue*', the bacterium responsible for yaws [3]. The genus *Borrelia* is a member of the family *Spirochaetaceae* and through convention is divided into three major clades: Lyme disease/Borreliosis (LB) caused by members of the *Borrelia burgdorferi* *sensu lato* complex, the Relapsing Fever (RF) borreliae and the Reptile-associated (REP) borreliae [4]. The LB borreliae currently comprise over 20 recognized species including the primary Northern hemisphere Lyme-disease-causing agents *Borrelia afzelii*, *Borrelia bavariensis*, *Borrelia burgdorferi* *sensu stricto*, and *Borrelia garinii*, along with a newly described genospecies ‘*Candidatus* Borrelia mayonii’ that causes LB in the upper Midwestern USA [5–8]. Members of the LB group are vectored by hard ticks (family Ixodidae), with the pathogenic species commonly transmitted to humans and other animals by ticks within the *Ixodes ricinus* complex: *Ixodes ricinus* in Europe, *Ixodes persulcatus* in Europe and Asia, and *Ixodes pacificus* and *Ixodes scapularis* in USA [7]. These pathogenic spirochaetes are dependent on wildlife, particularly rodents and birds, which act as asymptomatic reservoir hosts that maintain their life cycles and transmission [9].

Spirochaetes within the RF group have been reported throughout a number of continents, including Africa [10], Eurasia [11] and North America [12]. These borreliae, in contrast to the LB group, are generally transmitted by soft ticks (family Argasidae), with the exceptions of *Borrelia miyamotoi* identified in *I. persulcatus* [13], *I. ricinus* [14], *I. pacificus* [15] and *I. scapularis* [16]; *‘Borrelia lonestari*' in *Amblyomma americanum* [17]; *Borrelia theileri* in *Rhipicephalus microplus* [18]; and ‘*Candidatus* Borrelia texasensis’ in *Dermacentor variabilis* [19].

The third major clade of this genus, the REP borreliae, was identified after the discovery of *Borrelia turcica* in *Hyalomma aegyptium* ticks collected from tortoises in Turkey [20, 21], followed by subsequent addition of REP-related species of the genus *Borrelia* identified in various reptiles [4, 22]. While the LB and RF spirochaetes consist of zoonotic pathogens and are of significant public health concern in many countries, the pathogenicity and zoonotic potential of the REP group are not yet known.

Although LB borreliae have never been identified in Australian ticks, wildlife or people [23], two RF borreliae are recognized: *B. theileri* and *Borrelia anserina* that are transmitted by *Rhipicephalus (Boophilus) australis* [24, 25] and *Argas persicus* [26, 27], respectively. In addition, ‘*Borrelia queenslandica*' was the first species of the genus *Borrelia* to be reported from native long-haired rats, *Rattus villosissimus*, in north-west Queensland, Australia. While the soft tick, *Ornithodorus gurneyi*, was considered to be the vector of this species due to its presence in the region, transmission experiments were not successful, and molecular characterization was never conducted to reliably identify the species of the genus *Borrelia* [28, 29].

Sequence analysis of multiple loci offers the advantage over morphological characterization of being highly discriminatory, therefore serving as a reliable method for accurate identification, characterization and population, and epidemiological analyses in numerous bacterial studies [30, 31]. This technique was first used on *Borrelia* *burgdorferi* in 2008 [32] and has become an increasingly common technique for taxonomic and epidemiological studies of this genus [33–36].

Recently, a novel species of the genus *Borrelia* was detected in a number of echidna ticks, *Bothriocroton concolor*, collected [37]. An additional representative was detected in an *Ixodes holocyclus* tick [38]. Based on the 16S rRNA (1097 bp) and flagellin (*flaB*, 400 bp) gene phylogenetic analyses, this species of the genus *Borrelia* formed a distinct clade from other well-described borreliae, indicating this organism, designated ‘*Borrelia* sp. Aus’, to be unique [37]. In the present study, we conducted sequence analyses of the *flaB*, *groEL*, *glpQ* and *gyrB* genes, in addition to the 16S rRNA and short *flaB* loci reported previously [37], to study the relationship of this novel species to other of the genus *Borrelia*. Phylogenetic analysis confirmed its species status, and we hereby propose to designate this species as ‘*Candidatus* Borrelia tachyglossi’.

This study was conducted under the compliance of the *Australian Code for the Responsible Conduct of Research, 2007* and *Australian Code for the Care and Use of Animals for Scientific Purposes, 2013*. Tick collection was carried out opportunistically with the approval from the Murdoch University Animal Ethics Committee.

Genomic DNA was extracted previously from 97 *Bothriocroton* *concolor* ticks, with 38 (39 %) ticks testing positive at the *Borrelia*-specific *flaB* locus [37]. These 38 *Borrelia*-positive ticks were included in this study. Nested- and hemi-nested PCRs were conducted using primers targeting the housekeeping genes *flaB* (763 bp), *groEL* (1537 bp), *glpQ* (874 bp) and *gyrB* (1702 bp) (Table S1, available in the online Supplementary Material). Primers were designed to amplify short fragments of each gene with overlapping regions in order to obtain maximum coverage of the genes analysed for accurate characterization. PCR cycling conditions were as described by Loh *et al*. [37] (see Table S1 for respective annealing temperatures), with the exception of *groEL* primers: initial denaturation at 95 °C for 5 mins, 35 cycles of denaturation at 95 °C for 30 s, annealing at 48 °C for 40 s, extension at 72 °C for 2 mins, and a final extension at 72 °C for 7 mins. Amplification products of the targeted DNA products were electrophoresed in 1–2 % agarose gel with blue light using safe (Invitrogen), and positive samples were purified and sent for Sanger sequencing.

DNA sequences generated at *flaB*, *groEL*, *glpQ* and *gyrB* loci were aligned and analysed together with sequences representing species of the genus *Borrelia* retrieved from GenBank. All sequences were aligned using mafft v7.017 [39] and then refined using muscle [40]. The best-fit model for each locus was assessed using mega6 [41] and was selected based on the Bayesian Information Criterion (BIC). Bayesian phylogenetic reconstructions using sequence alignments of all four loci were generated using MrBayes 3.2.6 [42], and concatenated phylogenetic reconstructions using concatenated sequence alignments were produced using the CIPRES Science Gateway V.3.3 [43]. GTR and HKY substitution models were selected, with gamma categories of five, MCMC length of 1 100 000, burn-in length of 10 000 and subsampling frequency of 200.

In the present study, *Borrelia*-specific *flaB* (763 bp), *groEL* (1537 bp), *gyrB* (1702 bp) and *glpQ* (874 bp) DNA sequences were successfully amplified and sequenced from 38 *Borrelia*-positive *Bothriocroton* *concolor* ticks described by Loh *et al*. [37]. Thirty samples were positive for all *flaB* fragments; 24 samples were positive for all *glpQ* fragments; 13 samples were positive for all *groEL* fragments; and 10 samples were positive for all *gyrB* fragments.

Previously, three closely related genotypes were distinguished in the *Bothriocroton* *concolor* ticks using the 16S rRNA gene sequences, tentatively given the designations *Borrelia* sp. Aus A, *Borrelia* sp. Aus B and *Borrelia* sp. Aus C [37]. However, in the present study, these genotypes are referred as ‘*Candidatus* Borrelia tachyglossi’ genotypes A, B and C, respectively. At the *flaB* locus, genotypes A, B and C consisted of nine, three and five identical samples, respectively. The *flaB* gene alignment (787 bp) between ‘*Candidatus* Borrelia tachyglossi’ genotypes and other described species of the genus *Borrelia* showed that the novel genotypes shared highest percentage sequence identities with *Borrelia turcica* from the REP group (87.9–88 %); similarities to the LB *Borrelia* group ranged from 82.1–83.2 %; with the least similarity to *Borrelia hispanica* from the RF group (79.8–80 %). The percentage identities within the ‘*Candidatus* Borrelia tachyglossi’ genotypes ranged from 99.6–99.9 %. The percentage nucleotide identities between ‘*Candidatus* Borrelia tachyglossi’ genotypes and *Borreliaturcica* (87.9–88 %) were higher than that between *Borreliaturcica* and *Borrelia hermsii* (86.7 %). In contrast, the percentage nucleotide identities between ‘*Candidatus* Borrelia tachyglossi’ genotypes and *Borreliahermsii* (84.6–84.8 %) were lower than that between *Borreliaturcica* and *Borreliahermsii* (86.7 %) (Table S2). Phylogenetic analyses of the *flaB* gene locus showed that the ‘*Candidatus* Borrelia tachyglossi’ genotypes clustered most closely with *Borreliaturcica* with a high posterior probability (Fig. 1).

**Fig. 1.**
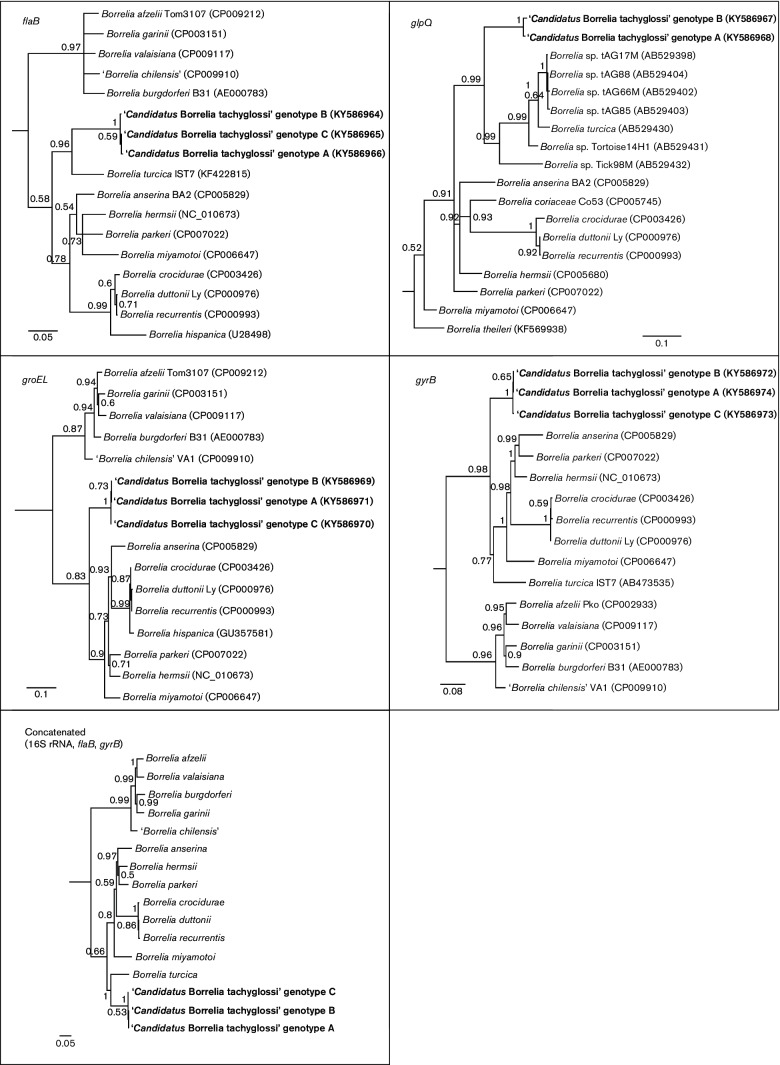
Phylogenetic reconstructions based on *flaB*, *groEL*, *glpQ* and *gyrB* gene sequences, and concatenated gene sequences of ‘*Candidatus* Borrelia tachyglossi’ genotypes A, B, and C identified in *Bothriocroton* *concolor* ticks from echidnas. *Brachyspirapilosicoli* (AY241832), *Treponema pallidum* (NZ_CP010566), *Escherichia coli* (X56907) and *Spirochaeta lutea* (JNUP01000064) were used as outgroups for each gene, respectively. Sequences determined in this study are indicated in bold type. Node labels represent posterior probabilities. Bars, substitutions per nucleotide position.

At the *glpQ* locus, genotypes A and B consisted of eight and two identical samples respectively. The amplification of this gene was not successful for genotype C. The *glpQ* nucleotide alignment (947 bp) between ‘*Candidatus* Borrelia tachyglossi’ genotypes exhibited 83.9–85.7 % similarity with the REP species of the genus *Borrelia* and lower similarities (80.6–84.8 %) with the RF species of the genus *Borrelia*. The percentage similarity within ‘*Candidatus* Borrelia tachyglossi’ genotypes was 98.6 %. Phylogenetic analysis confirmed the closer relationship of the ‘*Candidatus* Borrelia tachyglossi’ genotypes from the present study with the REP *Borrelia* group (100 % bootstrap support) (Fig. 1). The percentage nucleotide identities between ‘*Candidatus* Borrelia tachyglossi’ genotypes and *Borrelia* *turcica* (84.1–84.2 %) and *Borrelia coriaceae* (RF) (84.1–84.8 %) were higher than that between *Borrelia* *turcica* and *Borrelia* *coriaceae* (83.3 %) (Table S3).

At the *groEL* locus, ‘*Candidatus* Borrelia tachyglossi’ genotypes A and B were identical, and both shared 99.9 % similarities with ‘*Candidatus* Borrelia tachyglossi’ genotype C. Nucleotide alignment (1540 bp) between ‘*Candidatus* Borrelia tachyglossi’ genotypes and other described species of the genus *Borrelia* showed that the novel isolates had the least similarity with the LB group (83.9–85 %) and were most similar to *Borrelia hermsii* from the RF group (89.3–89.4 %). Phylogenetic analysis of the *groEL* locus showed that ‘*Candidatus* Borrelia tachyglossi’ genotypes from the present study clustered with the RF group with strong posterior probability support (Fig. 1). The percentage nucleotide identities between ‘*Candidatus* Borrelia tachyglossi’ genotypes and *Borreliahermsii* (89.3–89.4 %) were higher than that between *Borreliahermsii* and *Borreliaburgdorferi* (84.8 %); whereas the percentage identity between ‘*Candidatus* Borrelia tachyglossi’ genotypes and *Borreliaburgdorferi* (84.3 %) was slightly lower (Table S4). The genetic distance between ‘*Candidatus* Borrelia tachyglossi’ and *Borreliaturcica* at this locus remains to be determined until the *groEL* gene is characterized in *Borreliaturcica*.

At the *gyrB* locus, ‘*Candidatus* *Borrelia* tachyglossi’ genotypes A and B were identical. Nucleotide alignment (1712 bp) revealed that the ‘*Candidatus* Borrelia tachyglossi’ genotypes exhibited 80.8–82.0 %, 80.8–82.0 % and 84.8–88.2 % sequence similarity with the LB Borreliae, the RF group and *Borrelia* *turcica*, respectively. The percentage similarities within ‘*Candidatus* Borrelia tachyglossi’ genotypes ranged from 99.6 to 99.9 %. Phylogenetic analysis showed that ‘*Candidatus* *Borrelia* tachyglossi’ genotypes formed their own monophyletic clade, separate from *Borrelia* *turcica* and the RF group, with high bootstrap support (98 %) (Fig. 1). The percentage nucleotide identities between ‘*Candidatus* Borrelia tachyglossi’ genotypes and *Borrelia* *turcica* (87.8–88 %) and *Borrelia* *hermsii* (88.1–88.2 %) were higher than that between *Borrelia* *turcica* and *Borrelia* *hermsii* (86.7 %) (Table S5).

A Bayesian phylogenetic tree reconstructed using the concatenated alignment (3585 bp) consisting of three genes, 16S rRNA, *flaB* and *gyrB*, available for each of the main borreliae (Fig. 1), illustrated that the ‘*Candidatus* Borrelia tachyglossi’ genotypes from *Bothriocroton* *concolor* ticks grouped separately, with *Borrelia* *turcica* as the closest relative (91.1–91.2 % nucleotide identities) (Table S6). Likewise, the concatenated alignment (5154 bp), which excluded *Borrelia* *turcica* (REP), based on the four loci amplified in the present study (16S rRNA, *flaB*, *groEL* and *gyrB*) also produced a similar tree topology with ‘*Candidatus* *Borrelia* tachyglossi’ genotypes forming a monophyletic clade supported by high posterior probabilities (Fig. S1), sharing the highest percentage identities with *Borrelia* *hermsii* (90.3 %) (Table S7).

All phylogenetic trees reconstructed revealed similar topologies with the REP group species of the genus *Borrelia* as the closest sister clade. The recently established REP group has been detected in various reptiles from several countries and in ticks that parasitise them [4, 21]. The recent discovery of a novel species of the genus *Borrelia* in *Amblyomma varenense* collected from the reticulated python (*Python reticulatus*) showed that the species of the genus *Borrelia* identified clustered together with the REP-associated *Borrelia* group, along with *Borrelia* *turcica*, based on phylogenetic analyses of 16S rRNA and *flaB* genes [44]. However, the pathogenic potential of the species of the genus *Borrelia* belonging to the REP group is unknown.

A number of members within the genus *Borrelia* are well known to cause diseases in humans outside of Australia; nonetheless, borrelial tick-borne disease in humans still remains highly speculative and controversial in this country. ‘*Candidatus* *Borrelia* tachyglossi’ was first reported in 2016 [37], hence the urgency to further characterize this bacterium on the basis of multi-loci gene sequencing in order to confirm the taxonomic position of this new member in the genus *Borrelia*. Our results, based on sequence and phylogenetic characterization of multiple loci, provide conclusive evidence that ‘*Candidatus* *Borrelia* tachyglossi’, identified in *Bothriocroton* *concolor* ticks from echidnas, is distinct from other described species of the genus *Borrelia* and constitutes to a new clade in this genus.

Borrelial spirochaetes are well known to be associated closely with wildlife and utilize tick vectors to maintain a sylvatic life cycle [45, 46]. Australian wildlife are also known to be involved in spill-over of various zoonotic parasites [47]. Therefore, it is plausible that this bacterium is also likely to persist in the environment through circulation among native ticks and native mammalian (including marsupial) hosts. Unlike the Australian paralysis tick *I. holocyclus*, *Bothriocroton* *concolor* is a highly specialized tick, with echidnas as their primary host, and with a geographic distribution known only in Australia and Papua New Guinea [48]. ‘*Candidatus* Borrelia tachyglossi’ has previously been identified in one human-biting tick (*I. holocyclus*) removed from an echidna [38] and its prevalence in *I. holocyclus* or other human-biting ticks remains to be determined. The morphological characteristics and the pathogenicity of this bacterium are also unknown.

## Description of ‘*Candidatus* Borrelia tachyglossi’

‘*Candidatus* Borrelia tachyglossi′ (ta.chy.glos′si. N.L. gen. n. *tachyglossi* of *Tachyglossus aculeatus*, the monotreme host of the ticks in which the bacterium was first identified]).

Species can be differentiated from other borreliae based on sequence and phylogenetic analyses of five genomic loci (16S rRNA, *flaB*, *groEL*, *gyrB* and *glpQ*). Comparisons of the *flaB* gene sequences among the ‘*Candidatus* Borrelia tachyglossi’ genotypes showed two single nucleotide polymorphisms (SNPs) in genotype A at bases 485 and 519 (GenBank accession no. KY586966); and one SNP in genotype B at base 227 (KY586964). As for the *groEL* gene, analysis revealed two SNPs in genotype C at bases 656 and 1143 (KY586970). Analysis of the *gyrB* gene showed two SNPs in genotype B at bases 985 and 1416 (KY586972); and five SNPs in genotype C at bases 405, 757, 1075, 1093 and 1420 (KY586973). At the *glpQ* locus, genotype A (KY586968) and genotype B (KY586967) showed 12 base differences at bases 3, 43, 103, 112, 264, 515, 520, 530, 532, 702, 819 and 852.

The DNA G+C contents for 16S rRNA, *flaB*, *groEL*, *gyrB* and *glpQ* genes of ‘*Candidatus* Borrelia tachyglossi’ genotype A are 47.3, 40.9, 38.5, 33.8 and 34.1 mol%, respectively. The DA G+C contents for 16S rRNA, *flaB*, *groEL*, *gyrB* and *glpQ* genes of ‘*Candidatus* Borrelia tachyglossi’ genotype B are 47.2, 40.9, 38.5, 33.9 and 34.7 mol%, respectively. The DNA G+C contents for 16S rRNA, *flaB*, *groEL*, and *gyrB* genes of ‘*Candidatus* Borrelia tachyglossi’ genotype C are 47.2, 41, 38.5 and 34 mol%, respectively.

## Supplementary Data

268 Supplementary File 1

## References

[1] Baranton G, Old IG (1995). The spirochaetes: a different way of life. Bull Inst Pasteur.

[2] Ma DY, Giacani L, Centurión-Lara A (2015). The molecular epidemiology of *Treponema pallidum* subspecies *pallidum*. Sex Health.

[3] Mitjà O, Marks M, Konan DJP, Ayelo G, Gonzalez-Beiras C (2015). Global epidemiology of yaws: a systematic review. Lancet Glob Health.

[4] Takano A, Goka K, Une Y, Shimada Y, Fujita H (2010). Isolation and characterization of a novel *Borrelia* group of tick-borne borreliae from imported reptiles and their associated ticks. Environ Microbiol.

[5] Margos G, Wilske B, Sing A, Hizo-Teufel C, Cao WC (2013). *Borrelia bavariensis* sp. nov. is widely distributed in Europe and Asia. Int J Syst Evol Microbiol.

[6] Pritt BS, Mead PS, Johnson DKH, Neitzel DF, Respicio-Kingry LB (2016). Identification of a novel pathogenic *Borrelia* species causing Lyme borreliosis with unusually high spirochaetaemia: a descriptive study. Lancet Infect Dis.

[7] Rudenko N, Golovchenko M, Grubhoffer L, Oliver JH (2011). Updates on *Borrelia burgdorferi* sensu lato complex with respect to public health. Ticks Tick Borne Dis.

[8] Takano A, Nakao M, Masuzawa T, Takada N, Yano Y (2011). Multilocus sequence typing implicates rodents as the main reservoir host of human-pathogenic *Borrelia garinii* in japan. J Clin Microbiol.

[9] Gern L, Humair P-F, Gray JS, Kahl O, Lane RS, Stanek G (2002). Ecology of *Borrelia burgdorferi* sensu lato in Europe. Lyme Borreliosis: Biology, Epidemiology and Control.

[10] Trape JF, Diatta G, Arnathau C, Bitam I, Sarih M (2013). The epidemiology and geographic distribution of relapsing fever borreliosis in West and North Africa, with a review of the *Ornithodoros erraticus* complex (Acari: Ixodida). PLoS One.

[11] Assous MV, Wilamowski A (2009). Relapsing fever borreliosis in Eurasia—forgotten, but certainly not gone!. Clin Microbiol Infec.

[12] Schwan TG, Raffel SJ, Schrumpf ME, Webster LS, Marques AR (2009). Tick-borne relapsing fever and *Borrelia hermsii*, Los Angeles County, California, USA. Emerg Infect Dis.

[13] Fukunaga M, Takahashi Y, Tsuruta Y, Matsushita O, Ralph D (1995). Genetic and phenotypic analysis of *Borrelia miyamotoi* sp. nov., isolated from the ixodid tick *Ixodes persulcatus*, the vector for lyme disease in Japan. Int J Syst Bacteriol.

[14] Fraenkel CJ, Garpmo U, Berglund J (2002). Determination of novel *Borrelia* genospecies in Swedish *Ixodes ricinus* ticks. J Clin Microbiol.

[15] Mun J, Eisen RJ, Eisen L, Lane RS (2006). Detection of a *Borrelia miyamotoi* sensu lato relapsing-fever group spirochete from *Ixodes pacificus* in California. J Med Entomol.

[16] Scoles GA, Papero M, Beati L, Fish D (2001). A relapsing fever group spirochete transmitted by *Ixodes scapularis* ticks. Vector Borne Zoonotic Dis.

[17] Barbour AG, Maupin GO, Teltow GJ, Carter CJ, Piesman J (1996). Identification of an uncultivable Borrelia species in the hard tick *Amblyomma americanum*: possible agent of a Lyme disease-like illness. J Infect Dis.

[18] Smith RD, Miranpuri GS, Adams JH, Ahrens EH (1985). *Borrelia theileri*: isolation from ticks (*Boophilus microplus*) and tick-borne transmission between splenectomized calves. Am J Vet Res.

[19] Lin T, Gao L, Seyfang A, Oliver JH (2005). '*Candidatus* Borrelia texasensis', from the American dog tick *Dermacentor variabilis*. Int J Syst Evol Microbiol.

[20] Güner ES, Hashimoto N, Kadosaka T, Imai Y, Masuzawa T (2003). A novel, fast-growing *Borrelia* sp. isolated from the hard tick *Hyalomma aegyptium* in Turkey. Microbiology.

[21] Güner ES, Watanabe M, Hashimoto N, Kadosaka T, Kawamura Y (2004). *Borrelia turcica* sp. nov., isolated from the hard tick *Hyalomma aegyptium* in Turkey. Int J Syst Evol Microbiol.

[22] Takano A, Fujita H, Kadosaka T, Konnai S, Tajima T (2011). Characterization of reptile-associated *Borrelia* sp. in the vector tick, *Amblyomma geoemydae*, and its association with lyme disease and relapsing fever *Borrelia* spp. Environ Microbiol Rep.

[23] Chalada MJ, Stenos J, Bradbury RS (2016). Is there a Lyme-like disease in Australia? Summary of the findings to date. One Health.

[24] Mulhearn CR (1946). A note on two blood parasites of cattle (*Spirochaeta theileri* and *Bartonella bovis*) recorded for the first time in Australia. Aust Vet J.

[25] Callow LL, Hoyte HMD (1961). Transmission experiments using *babesia bigemina*, *Theileria mutans*, *Borrelia* sp. and the cattle tick. Aust Vet J.

[26] Gorrie CJR (1950). Vaccination against spirochaetosis in fowls. Aust Vet J.

[27] Petney TN, Andrews RH, McDiarmid LA, Dixon BR (2004). *Argas persicus* sensu stricto does occur in Australia. Parasitol Res.

[28] Pope JH, Carley JG (1956). Isolation of *Borrelia* from native rats in north-west Queensland. Aust J Science.

[29] Carley JG, Pope JH (1962). A new species of Borrelia (*B. queenslandica*) from *Rattus villosissimus* in Queensland. Aust J Exp Biol Med Sci.

[30] Enright MC, Spratt BG (1999). Multilocus sequence typing. Trends Microbiol.

[31] Urwin R, Maiden MC (2003). Multi-locus sequence typing: a tool for global epidemiology. Trends Microbiol.

[32] Margos G, Gatewood AG, Aanensen DM, Hanincová K, Terekhova D (2008). MLST of housekeeping genes captures geographic population structure and suggests a European origin of *Borrelia burgdorferi*. Proc Natl Acad Sci USA.

[33] Toledo A, Anda P, Escudero R, Larsson C, Bergstrom S (2010). Phylogenetic analysis of a virulent *Borrelia* species isolated from patients with relapsing fever. J Clin Microbiol.

[34] Schwan TG, Anderson JM, Lopez JE, Fischer RJ, Raffel SJ (2012). Endemic foci of the tick-borne relapsing fever spirochete *Borrelia crocidurae* in Mali, West Africa, and the potential for human infection. PLoS Negl Trop Dis.

[35] Jacquot M, Bisseux M, Abrial D, Marsot M, Ferquel E (2014). High-throughput sequence typing reveals genetic differentiation and host specialization among populations of the *Borrelia burgdorferi* species complex that infect rodents. PLoS One.

[36] Jungnick S, Margos G, Rieger M, Dzaferovic E, Bent SJ (2015). *Borrelia burgdorferi* sensu stricto and *Borrelia afzelii*: population structure and differential pathogenicity. Int J Med Microbiol.

[37] Loh SM, Gofton AW, Lo N, Gillett A, Ryan UM (2016). Novel *Borrelia* species detected in echidna ticks, *Bothriocroton concolor*, in Australia. Parasit Vectors.

[38] Gofton AW, Oskam CL, Lo N, Beninati T, Wei H (2015). Inhibition of the endosymbiont “*Candidatus* Midichloria mitochondrii” during 16S rRNA gene profiling reveals potential pathogens in *Ixodes* ticks from Australia. Parasit Vectors.

[39] Katoh K, Misawa K, Kuma K, Miyata T (2002). MAFFT: a novel method for rapid multiple sequence alignment based on fast Fourier transform. Nucleic Acids Res.

[40] Edgar RC (2004). MUSCLE: multiple sequence alignment with high accuracy and high throughput. Nucleic Acids Res.

[41] Tamura K, Stecher G, Peterson D, Filipski A, Kumar S (2013). MEGA6: molecular evolutionary genetics analysis version 6.0. Mol Biol Evol.

[42] Huelsenbeck JP, Ronquist F (2001). MRBAYES: bayesian inference of phylogenetic trees. Bioinformatics.

[43] Miller MA, Pfeiffer W, Schwartz T (2010). Creating the CIPRES Science Gateway for inference of large phylogenetic trees.. *Proceedings of the Gateway Computing Environments Workshop (GCE),* 14 November.

[44] Trinachartvanit W, Hirunkanokpun S, Sudsangiem R, Lijuan W, Boonkusol D (2016). *Borrelia* sp. phylogenetically different from lyme disease- and relapsing fever-related *Borrelia* spp. in *Amblyomma varanense* from *Python reticulatus*. Parasit Vectors.

[45] Nakao M, Miyamoto K, Fukunaga M (1994). Lyme disease spirochetes in japan: enzootic transmission cycles in birds, rodents, and Ixodes persulcatus ticks. J Infect Dis.

[46] Oliver JH, Lin T, Gao L, Clark KL, Banks CW (2003). An enzootic transmission cycle of lyme borreliosis spirochetes in the southeastern United States. Proc Natl Acad Sci USA.

[47] Thompson RC (2013). Parasite zoonoses and wildlife: one health, spillover and human activity. Int J Parasitol.

[48] Roberts FHS (1970). Australian Ticks.

