# Supplementary material for: Molecular characterization of ‘Candidatus Borrelia tachyglossi’ (family Spirochaetaceae) in echidna ticks, Bothriocroton concolor
Source: Int J Syst Evol Microbiol. 2017 May 5;67(4):1075–80. doi: 10.1099/ijsem.0.001929 (PMC5817193; doi:10.1099/ijsem.0.001929)
Supplement: Supplementary File 1 [file ijsem-67-1075-s001.pdf]

Molecular characterisation of '*Candidatus* Borrelia tachyglossi' (family Spirochaetaceae) in echidna ticks, *Bothriocroton concolor*

Supplementary Materials

The International Journal of Systematic and Evolutionary Microbiology

Siew-May Loh[1], Amber Gillett[2], Una Ryan[1], Peter Irwin[1], Charlotte Oskam[1*]

[1]Vector and Water-Borne Pathogen Research Group, School of Veterinary and Life Sciences, Murdoch University, Perth, Western Australia, Australia

[2]Australia Zoo Wildlife Hospital, Beerwah, Queensland, Australia

*Corresponding Author: c.oskam@murdoch.edu.au

Supplementary Table 1. Primers used for the amplification and sequencing of *flaB*, *glpQ*, *groEL*, and *gyrB* gene sequences.

Gene	Forward primer (5'-3')	Reverse primer (5'-3')	Annealing temperature (°C)	Expected size (bp)	References
flaB fragment 1 (Primary)	TCTGATGATGCTGCTGGTATGG	CAATCGCCTCATCCTGAT	50	439	This study
flaB fragment 1 (Secondary)	TCTGATGATGCTGCTGGTATGG	GAAGCTTGCGCACCAGTTAG	57	392	This study
flaB fragment 2 (Primary)	CTGAAGAGCTTGGAATGCAAC	AGGTACTTGATTTGCTTGTGC	52	545	[1]
flaB fragment 2 (Secondary)	CTGAAGAGCTTGGAATGCAAC	GCAATCATAGCCATTGCAGATTGT	52	526	[1, 2]
glpQ fragment 1 (Primary)	CATTAATTATAGCTCACAGAG	AACAAGCATTATCAATTTTCC	48	599	[1]
glpQ fragment 1 (Secondary)	AGTGGATATTTACCAGAACA	AACAAGCATTATCAATTTTCC	48	573	This study; [1]
glpQ fragment 2 (Primary)	GCACGACCCAGAAATTGACAC	GTTGCYCCYCCRCCAACTAT	57	894	This study
glpQ fragment 2 (Secondary)	CTGGCATAAGCAACAAGGCA	GTTGCYCCYCCRCCAACTAT	57	603	This study
groEL fragment 1 (Primary)	TGGCTAAGGACATATATTTA	GCTCCAACATTAATAACAGC	48	1,141	This study; [1]
groEL fragment 1 (Secondary)	GATGCKAGAAAGAGYYTGCT	GCTCCAACATTAATAACAGC	48	1,115	This study
groEL fragment 2 (Primary)	ATTGCTATACTTACTGGAGG	TTACATCATTCCCATTCCAG	48	768	[1]
groEL fragment 2 (Secondary)	GGTGTGCTTATTAGTGAGGA	TTACATCATTCCCATTCCAG	50	750	This study; [1]
gyrB fragment 1 (Primary)	ATTGAYGARGCYTTRGCRGG	GACCACTCCTGAAACCAGCA	57	727	This study
gyrB fragment 1 (Secondary)	ATTGAYGARGCYTTRGCRGG	CYTCYGTCCAYTTAAGTCCA	51	643	This study
gyrB fragment 2 (Primary)	CAARGAGRCTTAGRGAACTTGC	CCATCAACATCAGCATCAGC	57	949	This study; [3]
gyrB fragment 2 (Secondary)	CAARGAGRCTTAGRGAACTTGC	CCAACACCAGCWCCAAGAGA	57	880	This study
gyrB fragment 3 (Primary)	CTTTGGGAAACTACTATGAAYCCTG	ACATCCAGATTTACTACATCAAGYG	51	764	This study
gyrB fragment 3 (Secondary)	CTTTGGGAAACTACTATGAAYCCTG	GGTTCAACWTCATCYCCCAT	51	713	This study

Supplementary Table 2. Genetic distance matrix of partial *flaB* gene sequences showing percentage nucelotide identities between '*Candidatus* Borrelia tachyglossi' genotypes A, B, and C, other *Borrelia* species, and the outgroup, *Brachyspira pilosicoli*.

	**1**	**2**	**3**	**4**	**5**	**6**	**7**	**8**	**9**	**10**	**11**	**12**	**13**	**14**	**15**	**16**	**17**
**1. *Candidatus* Borrelia tachyglossi genotype B (KY586964)**																	
**2. Candidatus Borrelia tachyglossi genotype C (KY586965)**	99.9																
**3. Candidatus Borrelia tachyglossi genotype A (KY586966)**	99.6	99.7															
**4. *Borrelia turcica* IST7 (KF422815)**	88.0	87.9	87.9														
**5. *Borrelia anserina* BA2 (CP005829)**	85.9	86.0	85.8	87.2													
**6. *Borrelia hermsii* (NC_010673)**	84.7	84.8	84.6	86.7	92.9												
**7. *Borrelia parkeri* (CP007022)**	84.6	84.7	84.5	87.3	93.2	93.3											
**8. *Borrelia miyamotoi* (CP006647)**	82.3	82.4	82.3	85.1	91.9	91.1	91.7										
**9. *Borrelia crocidurae* (CP003426)**	83.3	83.2	83.2	86.1	90.1	89.0	90.3	89.0									
**10. *Borrelia duttonii* Ly (CP000976)**	82.8	82.6	82.6	85.8	90.1	88.8	90.3	89.0	98.8								
**11. *Borrelia recurrentis* (CP000993)**	82.6	82.5	82.5	85.8	90.1	88.8	90.3	89.0	98.8	99.7							
**12. *Borrelia hispanica* (U28498)**	80.0	79.9	79.9	82.3	87.1	85.3	86.7	85.2	93.1	93.9	93.8						
**13. *Borrelia afzelii* Tom3107 (CP009212)**	83.2	83.1	82.8	85.6	85.5	84.1	84.2	82.8	84.1	83.5	83.5	81.3					
**14. *Borrelia garinii* (CP003151)**	83.0	82.8	82.6	85.3	84.5	83.3	84.1	81.7	83.2	82.6	82.6	80.0	94.7				
**15. *Borrelia valaisiana* (CP009117)**	82.7	82.6	82.3	86.2	84.6	84.1	84.5	83.0	83.9	83.7	83.7	81.2	95.7	95.2			
**16. *Borrelia chilensis* (CP009910)**	83.1	83.0	82.7	86.0	84.7	83.7	84.3	83.0	83.7	83.5	83.3	81.4	95.1	93.8	94.9		
**17. *Borrelia burgdorferi* B31 (AE000783)**	82.4	82.3	82.1	85.7	84.3	83.3	83.7	82.0	83.7	83.5	83.4	81.4	94.4	93.6	94.7	94.9	
**18. *Brachyspira pilosicoli* (AY241832)**	46.5	46.7	46.7	46.8	47.8	47.3	47.2	47.0	46.7	46.6	46.5	45.8	46.3	47.4	46.9	47.4	47.2

Supplementary Table 3. Genetic distance matrix of partial *glpQ* gene sequences showing percentage nucelotide identities between '*Candidatus* Borrelia tachyglossi' genotypes A, B, and C, other *Borrelia* species, and the outgroup, *Escherichia coli*.

	**1**	**2**	**3**	**4**	**5**	**6**	**7**	**8**	**9**	**10**	**11**	**12**	**13**	**14**	**15**	**16**	**17**	**18**
**1. *Candidatus* Borrelia tachyglossi genotype B (KY586967)**																		
**2. *Candidatus* Borrelia tachyglossi genotype A (KY586968)**	98.6																	
**3. *Borrelia* sp. tAG17M (AB529398)**	84.3	84.4																
**4. *Borrelia* sp. tAG88 (AB529404)**	84.4	84.8	99.7															
**5. *Borrelia* sp. tAG66M (AB529402)**	84.1	84.4	99.2	99.5														
**6. *Borrelia* sp. tAG85 (AB529403)**	84.3	84.7	99.2	99.5	99.1													
**7. *Borrelia turcica* (AB529430)**	84.1	84.2	95.5	95.4	95.0	95.0												
**8. *Borrelia* sp. Tortoise14H1 (AB529431)**	85.4	85.7	94.1	94.2	93.7	93.7	93.6											
**9. *Borrelia* sp. Tick98M (AB529432)**	83.9	84.0	86.5	86.3	85.9	86.3	85.6	86.6										
**10. *Borrelia anserina* BA2 (CP005829)**	83.8	83.8	83.2	83.3	83.1	83.3	84.4	83.6	82.0									
**11. *Borrelia coriaceae* Co53 (CP005745)**	84.1	84.8	83.0	83.0	82.7	82.8	83.3	83.1	82.8	87.6								
**12. *Borrelia parkeri* (CP007022)**	84.6	84.6	82.4	82.3	82.0	82.3	84.1	83.0	83.3	89.6	89.2							
**13. *Borrelia hermsii* (CP005680)**	84.1	84.3	83.0	82.8	82.8	82.7	84.4	83.0	83.5	88.3	87.9	90.3						
**14. *Borrelia miyamotoi* (CP006647)**	83.1	82.8	83.1	82.7	82.5	82.7	83.3	82.7	83.2	87.0	86.7	87.9	88.2					
**15. *Borrelia theileri* (KF569938)**	80.5	80.8	82.2	81.8	81.6	81.8	81.8	80.8	81.7	84.4	85.4	85.0	85.9	90.6				
**16. *Borrelia crocidurae* (CP003426)**	80.8	80.9	80.4	80.5	80.3	80.5	80.5	80.7	81.1	84.4	84.6	84.6	84.8	83.2	82.6			
**17. *Borrelia duttonii* Ly (CP000976)**	80.7	81.0	80.3	80.4	80.2	80.2	80.4	81.0	80.9	84.1	84.9	84.4	85.0	83.2	82.0	97		
**18. *Borrelia recurrentis* (CP000993)**	80.5	80.9	80.2	80.3	80.1	80.1	80.3	80.9	81.0	84.0	85.0	84.6	85.1	83.1	82.2	97	99.7	
**19. *Escherichia coli* (X56907)**	54.0	54.1	55.2	55.3	55.1	55.3	55.8	54.5	54.9	56.4	56.3	56.2	54.9	55.4	55.8	54	54.5	54.4

Supplementary Table 4. Genetic distance matrix of partial *groEL* gene sequences showing percentage nucelotide identities between '*Candidatus* Borrelia tachyglossi' genotypes A, B, and C, other *Borrelia* species, and the outgroup, *Treponema pallidum*.

	**1**	**2**	**3**	**4**	**5**	**6**	**7**	**8**	**9**	**10**	**11**	**12**	**13**	**14**	**15**	**16**
**1. *Candidatus* Borrelia tachyglossi genotype B (KY586969)**																
**2. *Candidatus* Borrelia tachyglossi genotype A (KY586971)**	100															
**3. *Candidatus* Borrelia tachyglossi genotype C (KY586970)**	99.9	99.9														
**4. *Borrelia anserina* (CP005829)**	88.9	88.9	89.0													
**5. *Borrelia miyamoto*i (CP006647)**	88.8	88.8	88.9	91.6												
**6. *Borrelia parkeri* (CP007022)**	89.0	89.0	89.1	92.8	92.9											
**7. *Borrelia hermsii* (NC_010673)**	89.3	89.3	89.4	92.9	92.5	94.3										
**8. *Borrelia crocidurae* (CP003426)**	88.2	88.2	88.3	91.9	91.8	91.9	92.2									
**9. *Borrelia duttonii* Ly (CP000976)**	87.9	87.9	88.0	91.9	91.5	91.7	91.9	99.2								
**10. *Borrelia recurrentis* (CP000993)**	88.0	88.0	88.2	92.0	91.7	91.9	91.9	99.3	99.6							
**11. *Borrelia hispanica* (GU357581)**	87.6	87.6	87.8	91.6	91.1	91.7	91.6	98.2	98.0	98.0						
**12. *Borrelia afzelii* Tom3107 (CP009212)**	84.3	84.3	84.3	84.4	85.6	84.9	85.2	84.7	84.8	84.9	84.5					
**13. *Borrelia garinii* (CP003151)**	83.9	83.9	83.9	84.3	85.2	84.8	84.4	84.3	84.5	84.6	84.2	96.2				
**14. *Borrelia valaisiana* (CP009117)**	84.0	84.0	83.9	84.3	85.4	84.6	84.8	84.4	84.5	84.6	84.3	96.1	95.7			
**15. *Borrelia burgdorferi* B31 (AE000783)**	84.3	84.3	84.3	84.4	85.4	85.0	84.8	84.5	84.6	84.7	84.1	95.8	95.6	94.5		
**16. *Borrelia chilensis* VA1 (CP009910)**	85.0	85.0	84.9	84.3	85.1	85.0	84.7	84.5	84.5	84.6	84.5	93.4	92.8	93.3	93.6	
**17. *Treponema pallidum* (NZ_CP010566)**	63.7	63.7	63.7	62.5	61.6	62.1	62.1	62.6	62.7	62.7	62.1	62.7	62.4	62.7	62.4	63.2

Supplementary Table 5. Genetic distance matrix of partial *gyrB* gene sequences showing percentage nucelotide identities between '*Candidatus* Borrelia tachyglossi' genotypes A, B, and C, other *Borrelia* species, and the outgroup, *Spirochaeta lutea*.

	**1**	**2**	**3**	**4**	**5**	**6**	**7**	**8**	**9**	**10**	**11**	**12**	**13**	**14**	**15**	**16**
**1. *Candidatus* Borrelia tachyglossi genotype B (KY586972)**																
**2. Candidatus Borrelia tachyglossi genotype A (KY586974)**	99.9															
**3. Candidatus Borrelia tachyglossi genotype C (KY586973)**	99.6	99.7														
**4. *Borrelia anserina* BA2 (CP005829)**	86.0	86.1	86.1													
**5. *Borrelia parkeri* (CP007022)**	86.9	87.0	86.8	91.1												
**6. *Borrelia hermsii* (NC_010673)**	88.1	88.2	88.2	90.7	92.8											
**7. *Borrelia miyamotoi* (CP006647)**	86.8	87.0	86.9	88.1	89.0	90.0										
**8. *Borrelia crocidurae* (CP003426)**	85.3	85.4	85.1	86.7	87.3	88.3	86.7									
**9. *Borrelia duttonii* Ly (CP000976)**	85.4	85.4	85.2	86.7	87.4	88.4	86.6	99.6								
**10. *Borrelia recurrentis* (CP000993)**	85.0	85.1	84.8	86.5	87.3	88.3	86.4	99.3	99.5							
**11. *Borrelia turcica* IST7 (AB473535)**	87.9	88.0	87.8	85.0	86.4	86.7	86.2	85.7	85.6	85.4						
**12. *Borrelia afzelii* Pko (CP002933)**	80.8	81.0	80.8	79.6	80.8	79.7	80.3	79.8	79.8	79.7	80.8					
**13. *Borrelia garinii* (CP003151)**	81.2	81.3	81.2	79.4	80.8	80.1	79.6	80.2	80.3	80.1	80.5	94.4				
**14. *Borrelia valaisiana* (CP009117)**	81.3	81.4	81.3	80.0	80.9	80.2	80.5	80.1	80.3	80.1	80.7	94.4	93.5			
**15. *Borrelia burgdorferi* B31 (AE000783)**	80.8	80.9	80.8	79.4	80.7	80.0	79.8	80.3	80.4	80.3	80.9	93.9	93.9	93.6		
**16. *Borrelia chilensis* VA1 (CP009910)**	81.9	82.0	82.0	80.1	81.6	81.2	80.6	81.0	80.9	80.7	80.7	92.9	92.9	92.7	92.7	
**17. *Spirochaeta lutea* (JNUP01000064)**	59.3	59.4	59.3	57.8	59.1	58.8	58.2	58.7	58.6	58.6	59.7	58.3	58.6	58.5	58.5	58.1

Supplementary Table 6. Genetic distance matrix of concatenated 16S, *flaB*, and *gyrB* gene sequences showing percentage nucelotide identities between '*Candidatus* Borrelia tachyglossi' genotypes A, B, and C, other *Borrelia* species, and the outgroup, *Spirochaeta lutea*.

	1	2	3	4	5	6	7	8	9	10	11	12	13	14	15	16
**1. *Candidatus* Borrelia tachyglossi genotype B**																
**2. *Candidatus* Borrelia tachyglossi genotype A**	99.8															
**3. *Candidatus* Borrelia tachyglossi genotype C**	99.7	99.8														
**4. *Borrelia turcica***	91.2	91.2	91.1													
**5. *Borrelia anserina***	89.6	89.6	89.6	89.3												
**6. *Borrelia hermsii***	90.7	90.8	90.8	90.2	93.6											
**7. *Borrelia parkeri***	89.9	90.0	89.9	90.1	93.9	94.9										
**8. *Borrelia miyamotoi***	89.2	89.3	89.3	89.3	91.9	92.8	92.4									
**9. *Borrelia crocidurae***	88.8	88.9	88.7	89.6	90.8	91.6	91.4	90.5								
**10. *Borrelia duttonii***	88.7	88.8	88.6	89.4	90.8	91.6	91.4	90.4	99.6							
**11. *Borrelia recurrentis***	88.5	88.5	88.4	89.3	90.7	91.4	91.3	90.3	99.3	99.6						
**12. *Borrelia afzelii***	86.0	86.0	86.0	86.7	86.0	85.7	86.4	85.6	85.7	85.6	85.4					
**13. *Borrelia valaisiana***	86.2	86.2	86.2	86.8	85.9	86.0	86.5	85.7	85.8	85.8	85.7	96.2				
**14. *Borrelia garinii***	86.2	86.2	86.2	86.4	85.5	85.8	86.3	85.1	85.6	85.6	85.4	95.8	95.5			
**15. *Borrelia burgdorferi***	85.8	85.8	85.9	86.8	85.6	85.8	86.3	85.1	85.9	86.0	85.8	95.6	95.5	95.5		
**16. *Borrelia chilensis***	86.7	86.7	86.7	86.9	86.2	86.6	87.0	85.9	86.3	86.3	86.0	95.2	95.1	94.9	95.1	
**17. *Spirochaeta lutea***	63.1	63.2	63.1	63.1	62.0	62.8	62.8	62.1	62.6	62.5	62.4	62.4	62.7	62.5	62.7	62.6

Supplementary Table 7. Genetic distance matrix of concatenated 16S, *flaB*, *groEL*, and *gyrB* gene sequences showing percentage nucelotide identities between '*Candidatus* Borrelia tachyglossi' genotypes A, B, and C, other *Borrelia* species, and the outgroup, *Spirochaeta lutea*.

	**1**	**2**	**3**	**4**	**5**	**6**	**7**	**8**	**9**	**10**	**11**	**12**	**13**	**14**	**15**
**1. *Candidatus* Borrelia tachyglossi genotype B**															
**2. *Candidatus* Borrelia tachyglossi genotype C**	99.8														
**3. *Candidatus* Borrelia tachyglossi genotype A**	99.9	99.8													
**4. *Borrelia anserina***	89.4	89.4	89.4												
**5. *Borrelia hermsi*i**	90.3	90.3	90.3	93.4											
**6. *Borrelia parkeri***	89.7	89.7	89.7	93.5	94.7										
**7. *Borrelia miyamotoi***	89.1	89.2	89.2	91.8	92.7	92.6									
**8. *Borrelia crocidurae***	88.6	88.6	88.6	91.1	91.8	91.6	90.9								
**9. *Borrelia duttonii***	88.5	88.4	88.5	91.1	91.7	91.5	90.8	99.5							
**10. *Borrelia recurrentis***	88.3	88.3	88.4	91.1	91.6	91.5	90.7	99.3	99.6						
**11. *Borrelia afzelii***	85.5	85.5	85.5	85.5	85.6	85.9	85.6	85.4	85.4	85.3					
**12. *Borrelia valaisiana***	85.5	85.5	85.5	85.5	85.7	85.9	85.6	85.4	85.4	85.3	96.2				
**13. *Borrelia garinii***	85.5	85.5	85.5	85.1	85.4	85.8	85.1	85.2	85.2	85.1	95.9	95.6			
**14. *Borrelia burgdorferi***	85.4	85.4	85.4	85.2	85.5	85.9	85.2	85.5	85.6	85.5	95.7	95.2	95.6		
**15. *Borrelia chilensis***	86.2	86.2	86.2	85.7	86.0	86.4	85.6	85.7	85.7	85.6	94.7	94.6	94.3	94.6	
**16. *Spirochaeta lutea***	62.9	62.9	63.0	62.2	62.6	62.5	62.0	62.6	62.5	62.4	62.5	62.7	62.5	62.7	62.7

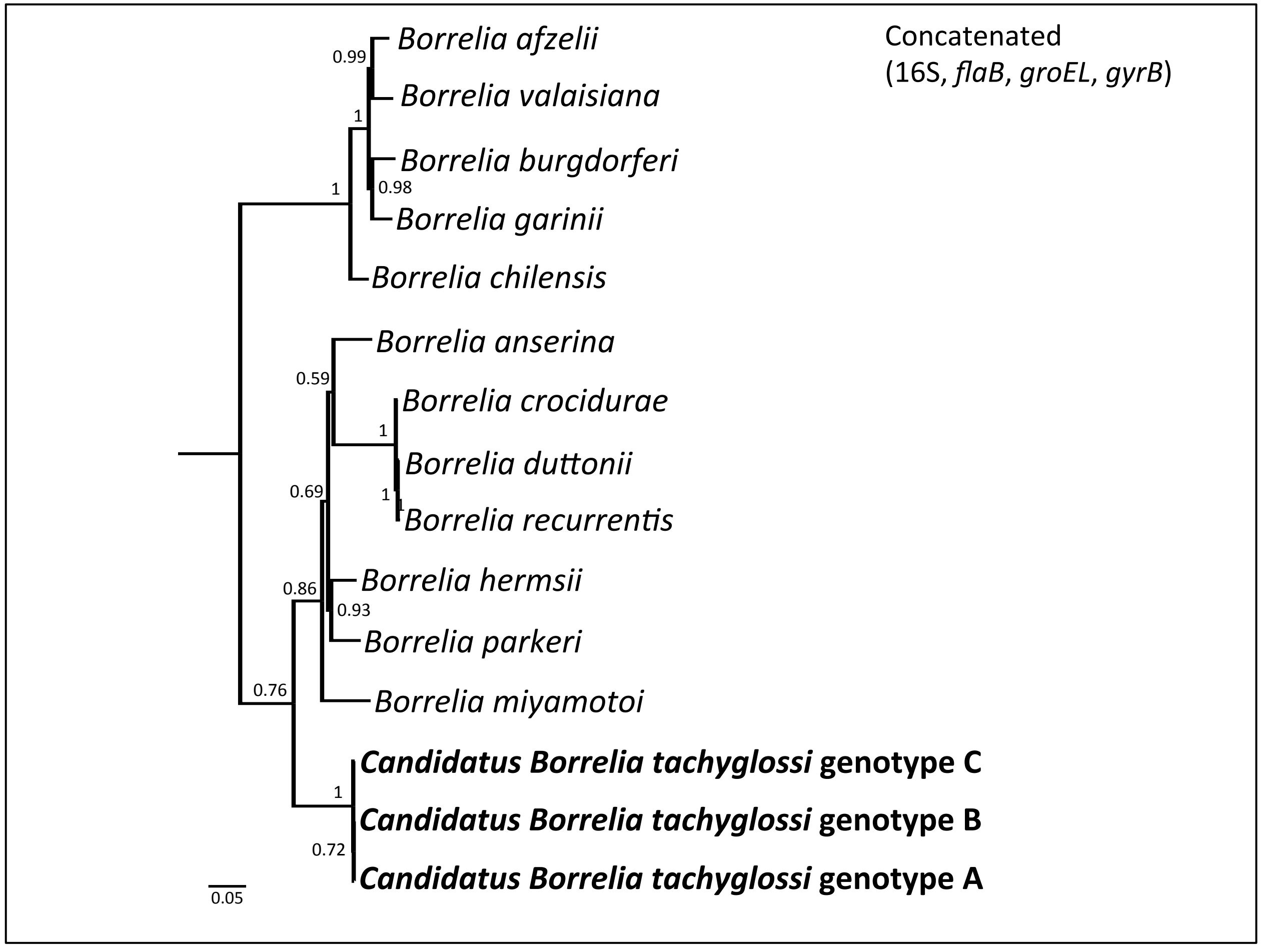

Supplementary Fig. 1. Phylogenetic reconstructions of concatenated 16S, *flaB*, *groEL*, and *gyrB* sequences of '*Candidatus* Borrelia tachyglossi' genotypes A, B, and C identified in *B. concolor* ticks from echidnas. *Spirochaeta lutea* was used as outgroup. Sequences in this study are indicated in bold.

References

1. **Toledo A, Anda P, Escudero R, Larsson C, Bergstrom S *et al.*** Phylogenetic analysis of a virulent *Borrelia* species isolated from patients with relapsing fever. *J Clin Microbiol* 2010; 48: 2484-2489.
2. **Barbour AG, Maupin GO, Teltow GJ, Carter CJ, Piesman J**. Identification of an uncultivable Borrelia species in the hard tick *Amblyomma americanum*: possible agent of a Lyme disease-like illness. *J Infect Dis* 1996; 173: 403-409.
3. **Schwan TG, Raffel SJ, Schrumpf ME, Policastro PF, Rawlings JA *et al.*** Phylogenetic Analysis of the Spirochetes Borrelia parkeri and Borrelia turicatae and the Potential for Tick-Borne Relapsing Fever in Florida. *J Clin Microbiol* 2005; 43: 3851-3859.
